# Differential responses of the rhizosphere microbiome structure and soil metabolites in tea (*Camellia sinensis*) upon application of cow manure

**DOI:** 10.1186/s12866-022-02470-9

**Published:** 2022-02-14

**Authors:** Litao Sun, Yu Wang, Dexin Ma, Linlin Wang, Xiaomei Zhang, Yiqian Ding, Kai Fan, Ze Xu, Changbo Yuan, Houzhen Jia, Yonglin Ren, Zhaotang Ding

**Affiliations:** 1 Tea Research Institute, Qingdao Agricultural University, Qingdao, China; 2 College of Science, Health, Engineering and Education, Murdoch University, Perth, WA Australia; 3 College of Communication, Qingdao Agricultural University, Qingdao, China; 4 College of Resource and Environment, Qingdao Agricultural University, Qingdao, China; 5 Tea Research Institute, Chongqing Academy of Agricultural Sciences, Chongqing, China; 6 Tea Research Institute, Shandong Academy of Agricultural Sciences, Jinan, China

**Keywords:** Rhizosphere, Bacterial microbiome, Metabolome, Fertilizer, Organic

## Abstract

**Background:**

The rhizosphere is the narrow zone of soil immediately surrounding the root, and it is a critical hotspot of microbial activity, strongly influencing the physiology and development of plants. For analyzing the relationship between the microbiome and metabolome in the rhizosphere of tea (*Camellia sinensis*) plants, the bacterial composition and its correlation to soil metabolites were investigated under three different fertilization treatments (unfertilized, urea, cow manure) in different growing seasons (spring, early and late summer).

**Results:**

The bacterial phyla *Proteobacteria*, *Bacteroidetes*, *Acidobacteria* and *Actinobacteria* dominated the rhizosphere of tea plants regardless of the sampling time. These indicated that the compositional shift was associated with different fertilizer/manure treatments as well as the sampling time. However, the relative abundance of these enriched bacteria varied under the three different fertilizer regimes. Most of the enriched metabolic pathways stimulated by different fertilizer application were all related to sugars, amino acids fatty acids and alkaloids metabolism. Organic acids and fatty acids were potential metabolites mediating the plant-bacteria interaction in the rhizosphere. Bacteria in the genera *Proteiniphilum*, *Fermentimonas* and *Pseudomonas* in spring, *Saccharimonadales* and *Gaiellales* in early summer, *Acidobacteriales* and *Gaiellales* in late summer regulated relative contents of organic and fatty acids.

**Conclusion:**

This study documents the profound changes to the rhizosphere microbiome and bacterially derived metabolites under different fertilizer regimes and provides a conceptual framework towards improving the performance of tea plantations.

**Supplementary Information:**

The online version contains supplementary material available at 10.1186/s12866-022-02470-9.

## Introduction

The composition of the microbiota inhabiting the rhizosphere is a major determinant of plant growth and productivity. More specifically, soil microbial communities provide critical services to plants, such as nutrient bioavailability and suppression of phytopathogens, and may directly influence crop quality [1–3]. Complex and dynamic interactions between plants and networks of microorganisms are intricate and challenging to study [4, 5]. Several specific benefits have been identified, including the presence of secretory systems, adhesion, metal detoxification and iron dissolution, all of which were associated with the rhizosphere enrichment [6]. Studying plant–microbe interactions will not only provide a better understanding of the relationship between soil metabolites and microbiomes, but it will inform management of such relationships for sustainable agricultural production.

Tea plant [*Camellia sinensis* (L.) O. Kuntze] is an evergreen leafy shrub whose leaves are used in the manufacture of beverages. Edaphic conditions are fundamental to the resource capture and productivity of tea plants. Traditionally, the supplement of inorganic or organic fertilizers for tea plantations increases soil fertility and tea quality [7]. Organic fertilizers can promote higher biomass and soil-borne bacteria and fungi diversity than chemical fertilizers [8]. Soil microbes and the yield and quality of plants show a strong positive correlation with each other [9]. In turn, plants support microbial communities by transferring 5–20% of photosynthetic carbon to the rhizosphere through the roots [10]. Our previous research has presented a detailed characterization of the rhizosphere microbiomes under two different fertilizer/manure applications and the important environmental properties in the rhizosphere soil were identified affecting the soil bacterial community structure in tea plantation [11]. However, the mechanism among the plant–microbiome interaction based on the metabolic level and the chemical driver under different fertilizer/manure applications is not well understood.

Here, based on our previous study in which the bacterial composition in the rhizosphere soil were identified, we further analyzed the metabolome of rhizosphere soil under three different fertilizer treatments (T1, unfertilized; T2, urea; T3, cow manure) applied in March, June and August (spring, early summer, late summer) using gas chromatography-mass spectrometry (GC–MS). The objectives were to: 1) identify the differential metabolites in the rhizosphere soil influenced by two different fertilizer/manure applications in three different growing seasons (spring, early and late summer), and 2) evaluate the relationship between the composition of bacterial communities and differential metabolites. The ultimate aim is to optimize the soil microbiome and identify specific compounds linking the rhizosphere with improved development and performance of tea plantations.

## Results

### Influence of treatment and sampling time on chlorophyll content of tea leaves

**Fig. 1:**
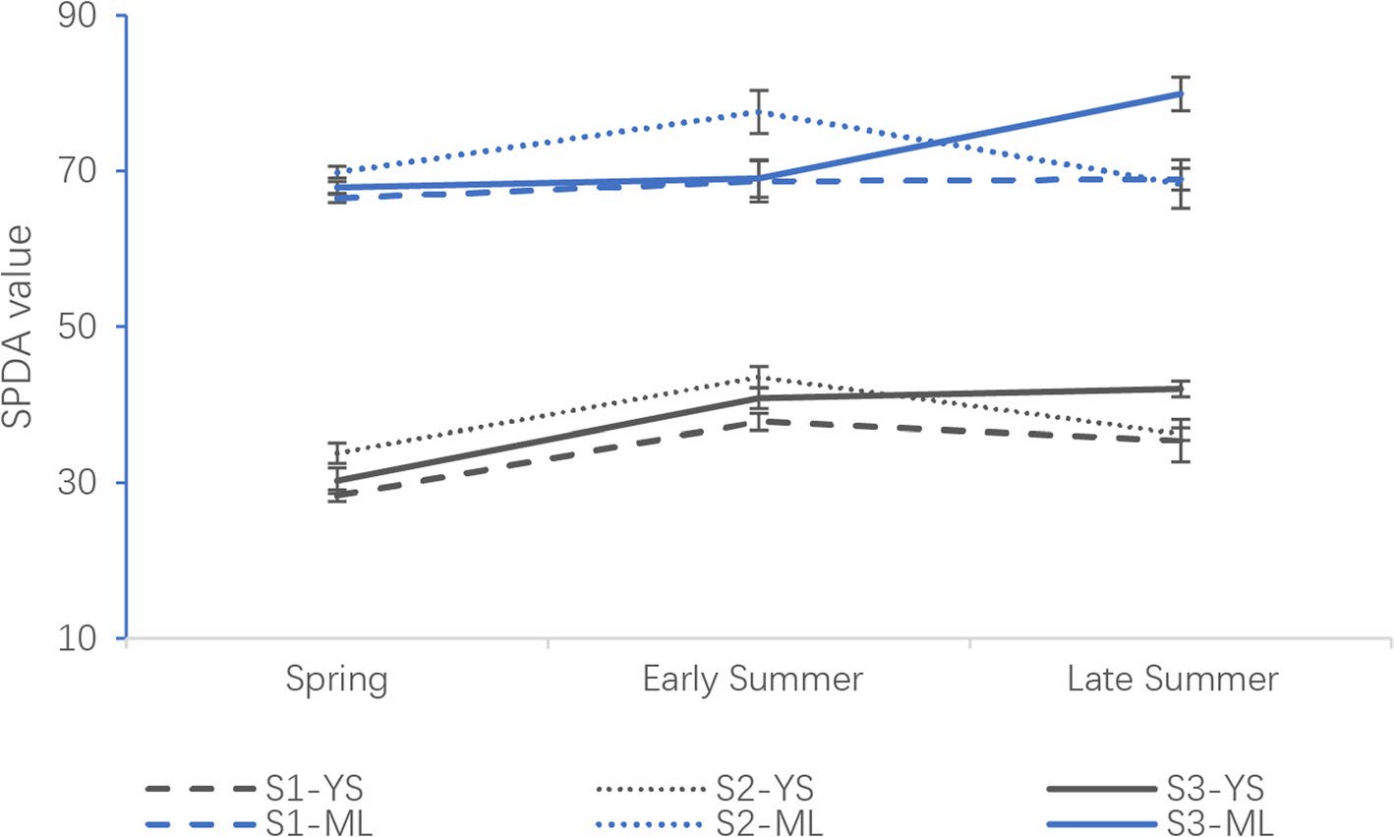
SPAD values of the young shoot (YS) and mature leaf (ML) under three different treatments in spring, early summer, and late summer

For evaluation of effect on the tea plant growth under the different fertilizer/manure treatments, the relative content of chlorophyll in tea leaves was analyzed. Similar trends were observed in young shoots (YS) and mature leaves (ML) with the same treatment (Fig. 1). In addition, the content of chlorophyll in ML was approximate double that of their YS counterparts. There was a slight increase of the content of chlorophyll in T3 from spring to late summer, while the content of chlorophyll in T1 and T2 increased in early summer and then decreased by late summer regardless in YS and ML. In spring and early summer, the content of chlorophyll in tea leaves in S2 was higher than that in S1 and S3.. In late summer, the content of chlorophyll in S3 increased to the maximum, which was higher than their counterparts in S1 and S2. Chlorophyll content was not significantly different between S1 and S2 in late summer.

### Soil microbiome composition under different fertilizer treatment s

**Fig. 2:**
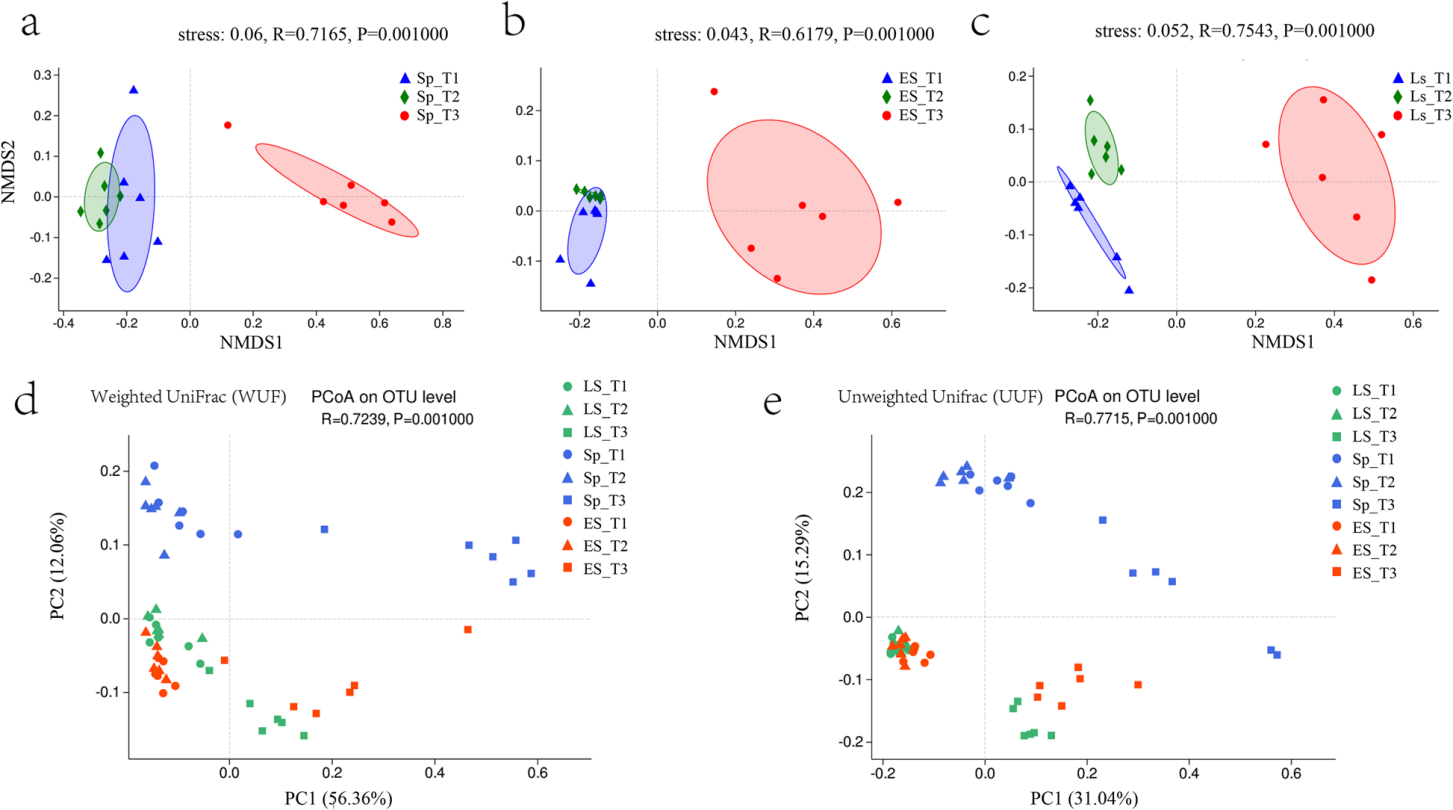
Differences in microbial community composition in the soil samples using NMDS and PCoA. (1) NMDS using the WUF metric indicates show separations at (**a**) spring (Sp), (**b**) early summer (ES), and (**c**) late summer (LS); (2) PCoA were performed with the Weighted UniFrac (WUF, **d**) and the Unweighted Unifrac (UUF, **e**)

Our previous study has shown that all of the samples shared the same taxa composition but differed in their relative abundances at the phylum level [11]. All samples were dominated (> 65%) by four phyla: *Proteobacteria*, *Bacteroidetes*, *Acidobacteria* and *Actinobacteria* (Figure S1, Table S2, S3). Here, to further determine the similarities and dissimilarities of soil microbial communities under different fertilizer treatment and different sampling time, beta diversity was calculated using Non-metric Multidimensional Scaling (NMDS) and Principal Coordinate Analysis (PCoA). All the stress values of NMDS were less than 0.01. The analysis revealed that the composition of soil microbial communities was influenced by different fertilizer/manure treatments, and the microbial community of T3 was statistically distinct from those of T1 and T2 (Fig. 2a, b and c). To further elucidate patterns of separation between microbial communities, unconstrained principal coordinate analyses were performed with the Weighted UniFrac (WUF) and the Unweighted Unifrac (UUF) (Fig. 2d and e). These indicated that the compositional shift was associated with different fertilizer/manure treatments as well as the sampling time. Both of the WUF and UUF PCoA described sampling time as having the second-largest source of variation (PC1:12.06%, WUF; PC1:16.29%, UUF; *R* > 0.7, *P* = 0.001) in rhizosphere microbial communities following the type of treatment (PC1:56.36%, WUF; PC1:30.04%, UUF; *R* > 0.7, *P* = 0.001). Consistently, the Permutational multivariate analysis of variance (PERMANOVA) corroborated that fertilizer/manure treatments contributed a larger source of variation within the microbiome when using a Bray Curtis distance metric (R2 = 0.312, *P* < 0.001; Table S4), compared to the contribution of sampling time (R2 = 0.246, *P* < 0.001; Table S4).

**Fig. 3:**
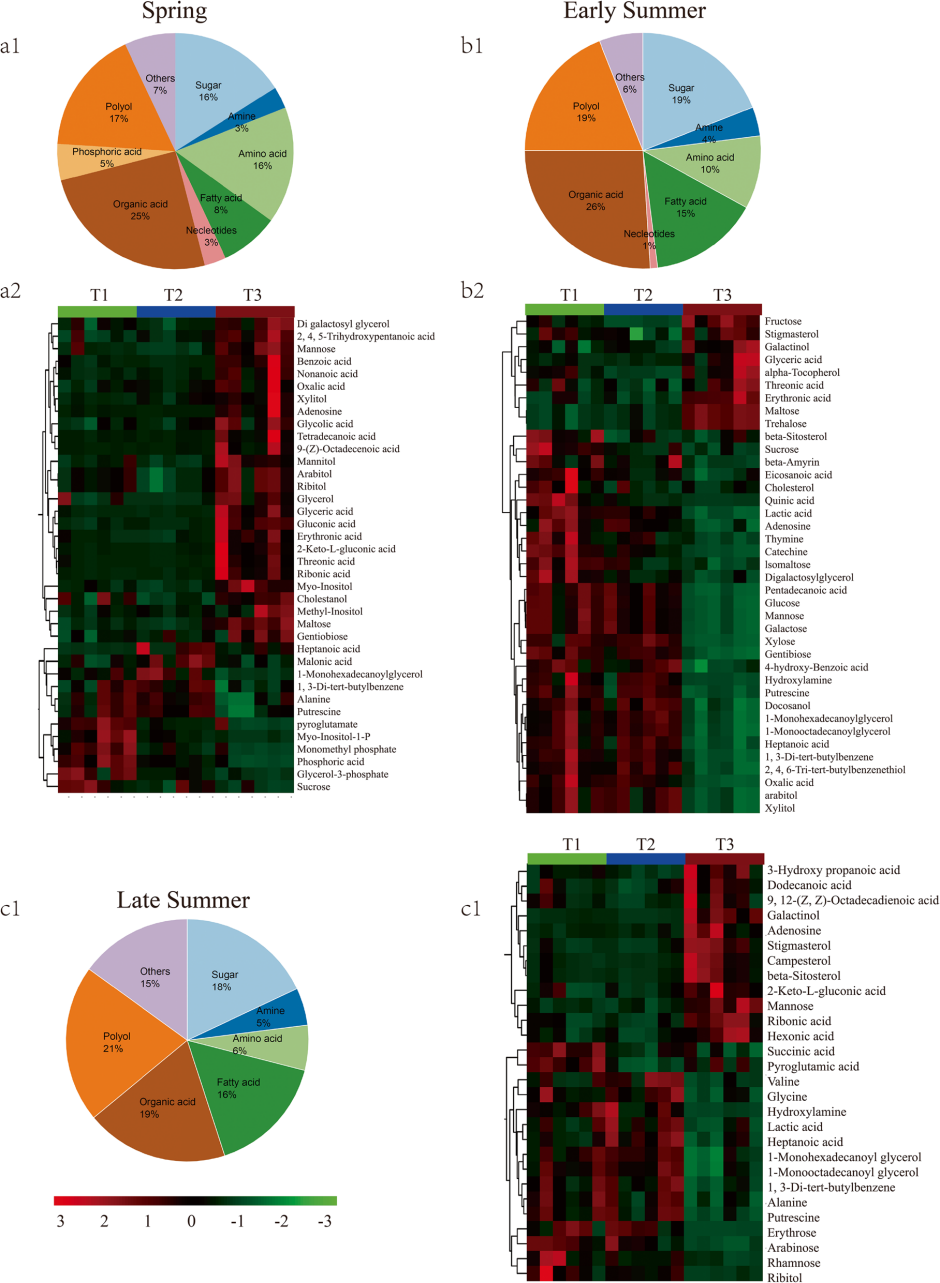
Metabolomics analysis of different fertilizer treatments in spring, early summer and late summer. a1, b1 and c1 (the pie chart) represent the categories and the percentages of soil metabolites based on the number of compounds; a2, b2 and c2 (the heat map) represent the differential relative content of common metabolites under different fertilizer treatments in spring, early summer and late summer, respectively. Six biological replicates for each soil type are displayed in separate stacked bars in each heat map

Bacterial communities were analyzed at the genus level to determine which genera of the *Proteobacteria* and *Actinobacteria* in T3 changed during the three sampling times. Thirty-two genera belonging to *Proteobacteria* significantly (*P* < 0.05) changed (Table S5). For example, the abundance of *Haliangium*, *Steroidobacter* and *Acidibacter* in T3 increased from spring to late summer, while *Pseudomonas* sharply decreased in the same period. Nine genera belonging to *Actinobacteria* significantly changed. For example, the abundance of *Acidothermus*, *Nocardioides* and *Mycobacterium* in T3 reached maximum abundance in early summer and then declined into late summer.

### Analysis of soil metabolites under different fertilizer treatments

Supervised Partial least Squares-discriminant Analysis (PLS-DA) was performed to investigate changes in soil metabolites with each fertilizer/manure treatment. PLS-DA score plots showed that the metabolites in T1, T2 and T3 were statistically separated from each sampling season (Figure S2). GC–MS analyses revealed 75 metabolites detected in spring, 73 in early summer and 62 in late summer (Table S6). All metabolites present in all three treatments, but the abundance of them has significant differences. The identified compounds included organic acids, polyols, amino acids, sugars, fatty acids, phosphoric acids, amines, nucleotides, and others (Fig. 3a1, b1 and c1). Specifically, organic acids showed the greatest changes in spring and early summer, accounting for 26% and 27% of the totals, respectively, followed by a decrease to 19% in late summer. Polyols and fatty acids increased from spring to late summer, while amino acids decreased over that period.

A heatmap with Hierarchical clustering analysis (HCA) was used to visualize and group differentially expressed metabolites among the three fertilizer/manure treatments. Thirty-eight differential metabolites were identified at the spring sampling, 28 in early summer, and 39 in late summer (Fig. 3a2, b2 and c2). In addition, all T3 samples clearly clustered together from the three different growing seasons, while T1 and T2 did not cluster in one group separately. It demonstrated that the application of cow manure considerable stimulated major changes in metabolism in the rhizosphere soil. Specifically, in comparison with T1 and T2, cow manure can mainly drive the accumulation of fatty acids (nonanoic acid, oxalic acid, tetradecanoic acid and octadecadienoic acid), organic acids (trihydroxy pentanoic acid, benzoic acid, glycolic acid, glyceric aicd, gluconic acid, erythronic acid and ribonic acid), alcohols (xylitol, mannitol, arabitol and ribitol) and mannose in spring; fatty acids (dodecanoic acid, octadecadienoic acid and hexenoic acid), organic acids (hydroxy propanoic acid, gluconic acid and ribonic acid), sterols (stigmasterol and campesterol) and mannose in early summer; sugars (fructose, maltose and trehalose), organic acids (glyceric aicd, threonic acid, and erythronic acid) and stigmasterol in late summer. Interestingly, the alanine had the lowest relative concentration in T3 compared with T1 and T2 in spring and early summer, while it could not be detected in late summer regardless of the different treatments.

### Analysis of metabolic pathway in the rhizosphere soil under different fertilizer treatments

**Fig. 4:**
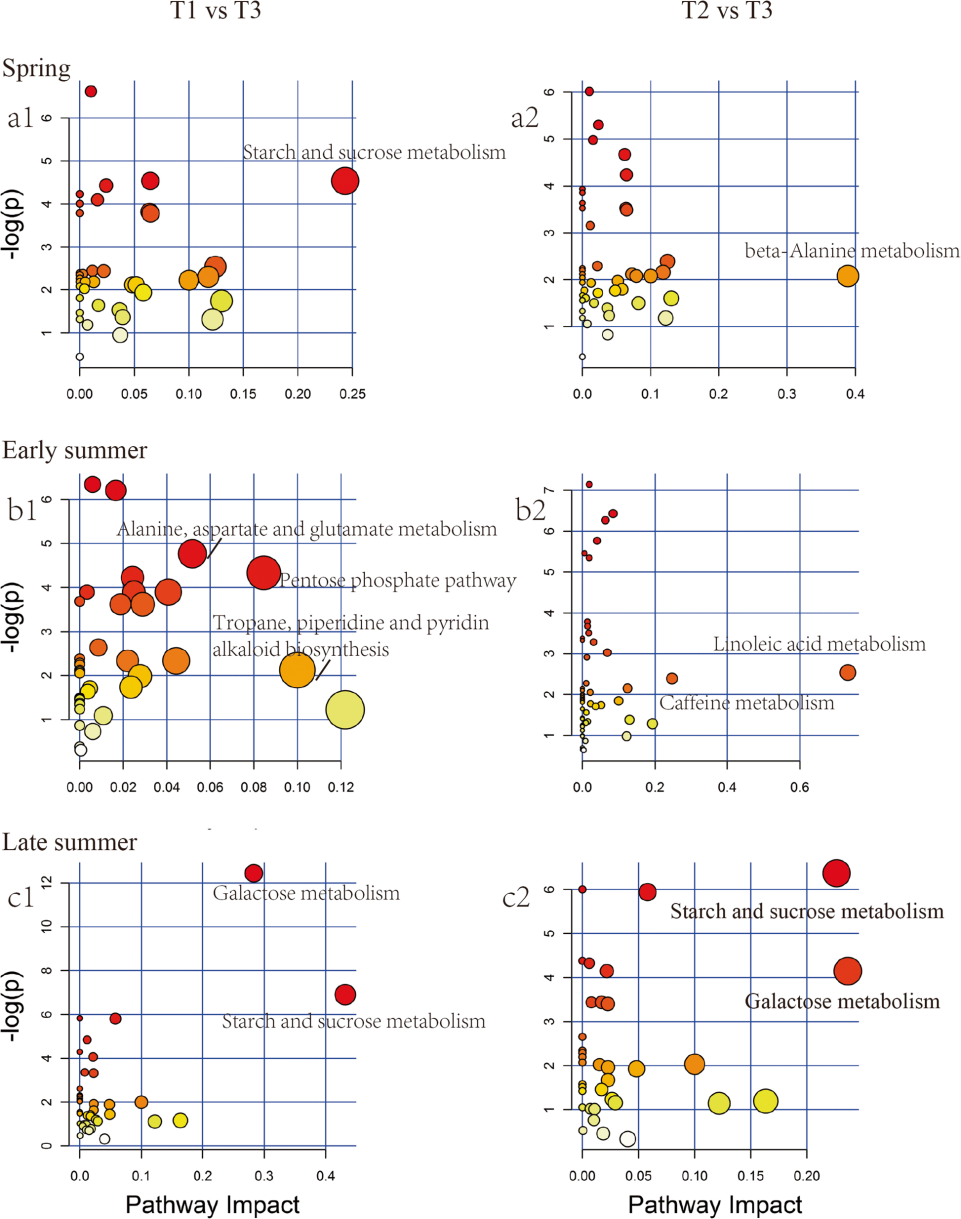
The enriched metabolomic pathways in the three rhizosphere soils were analyzed based on the KEGG dataset between T1 and T3, T2 and T3 in spring (a1 and a2, respectively), early summer (b1 and b2, respectively) and late summer (c1 and c2, respectively)

To further analyze the variation of the metabolites in the rhizosphere soil treated with different fertilizer treatments, the metabolic pathway was proposed in reference to the Kyoto Encyclopedia of Genes and Genomes database (KEGG). Several significantly enriched pathways (Impact > 0.1) were identified among different fertilizer application. As shown in Fig. 4, the enriched metabolic pathways in T3 compared with T1 were starch and sucrose metabolism in spring, alanine, aspartate and glutamate metabolism, pentose phosphate pathway and tropane, piperidine and pyridine alkaloid biosynthesis in early summer, galactose metabolism and starch and sucrose metabolism in late summer. In comparison to T2, the differential metabolic pathways in T3 were beta-alanine metabolism in spring, linoleic acid metabolism and caffeine metabolism in early summer, starch and sucrose metabolism and galactose metabolism in late summer. Notably, compared T3 with the other treatments, the most differentially enriched pathways were sugars and fatty acids related metabolisms. In addition, the metabolites in T3 showed considerable differences in late summer in starch and sucrose metabolism and galactose metabolism.

### Correlations between microbiota and metabolites under different fertilizer treatments

**Fig. 5:**
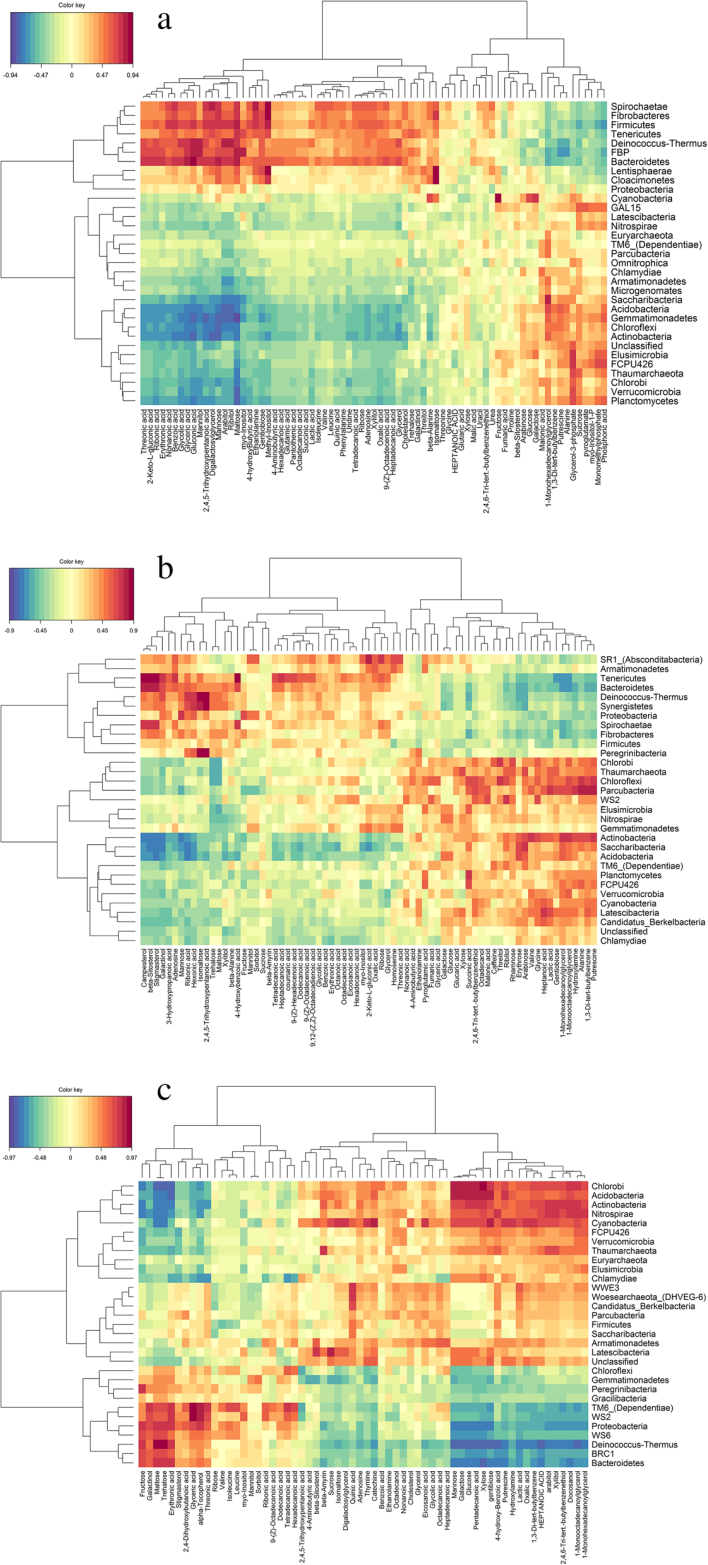
Correlation analyses of soil microbiome and metabolome. Hierarchical clustering of the soil metabolites based on the microbes of a phylum-level in spring (**a**), early summer (**b**), and late summer (**c**). Positive and negative correlations are represented by red and blue, respectively

To decipher the potential regulation effects between the microbiota and the metabolites in rhizosphere soil, the correlations between the soil microbial communities at the phylum level and metabolites were analyzed by Pearson correlation coefficients method and were visualized as a heatmap (Fig. 5). Based on the correlation analysis, six apparent groups were clustered between soil microbes and metabolites from three different growing seasons.

In spring (Fig. 5a), 10 bacterial phyla (*Proteobacteria, Bacteroidetes, Cyanobacteria*, etc.) clustered together showing a similar correlation. Specifically, they showed a positive correlation with most organic acids (threonic acid, 2-keto-L-gluconic acid, ribonic acid, etc.) and polyols (mannitol, digalactosycerol, arabitol, etc.), while there was a negative correlation to most phosphoric acids (phosphoric acid, monomethylphosphate, myo-inositol-1-p, etc.). In addition, 11 phyla (*Saccharibacteria*, Acidobacteria, *Chloroflexi,* etc.) showed similar patterns. They had positive correlations with most phosphoric acids, but negative correlations with all fatty acids (including nonanoic acid, hexadecanoic acid, octadecanoic acid, tetradecanoic acid, 9-(Z)-octadecenoic acid and heptadecanoic acid), all nucleotides (including adenosine and uridine) and most organic acids. In addition, the remaining bacterial phyla showed a similar correlation pattern but without obvious distinction among differential metabolites.

In early summer (Fig. 5b), 11 phyla (*Proteobacteria, Bacteroidetes, Absconditabacteria*, etc.) were clustered together showing a similar correlation profile. The metabolome associated with these bacteria included 13 metabolites with positive correlations, the three strongest of which were campesterol, beta-sitosterol and stigmasterol, and eight metabolites (1-monohexadecannoylglycerol, arabinose, erythrose, etc.) had a negative correlation with these bacteria. On the lower right of Fig. 5b, 12 phyla (*Cyanobacteria, Chloroflexi, Berkelbacteria*, etc.) clustered together showing a similar profile. The metabolome associated with these bacteria included 13 metabolites with positive correlations, the three strongest of which were putrescine, 1,3-di-tert-butylbenzene and alanine, and 29 metabolites had negative correlations, the three strongest of which were campesterol, beta-sitosterol and stigmasterol.

In late summer (Fig. 5c), 11 phyla (*Chlorobi*, *Acidobacteria*, *Actinobacteria*, *Cyanobacteria,* etc.) were clustered together showing a similar correlation profile. The metabolome associated with these bacteria included 22 metabolites with positive correlations, the three strongest of which were 1-monohexadecanoylglycerol, 1-monooctadecanoylglycerol and xylitol. Fifteen metabolites negatively correlated with these bacteria, the three strongest of which were fructose, maltose and trehalose. On the lower right of Fig. 5c, 11 phyla (*Proteobacteria, Chloroflexi, Bacteroidetes*, etc.) were clustered together showing a similar profile. The features of metabolome associated with these bacteria included 12 metabolites (maltose, trehalose, erythronic acid, etc.) with positive correlations, and 29 metabolites had a negative correlation with these bacteria, the three strongest of which were pentadecanoic acid, gentibiose and glucose.

**Fig. 6:**
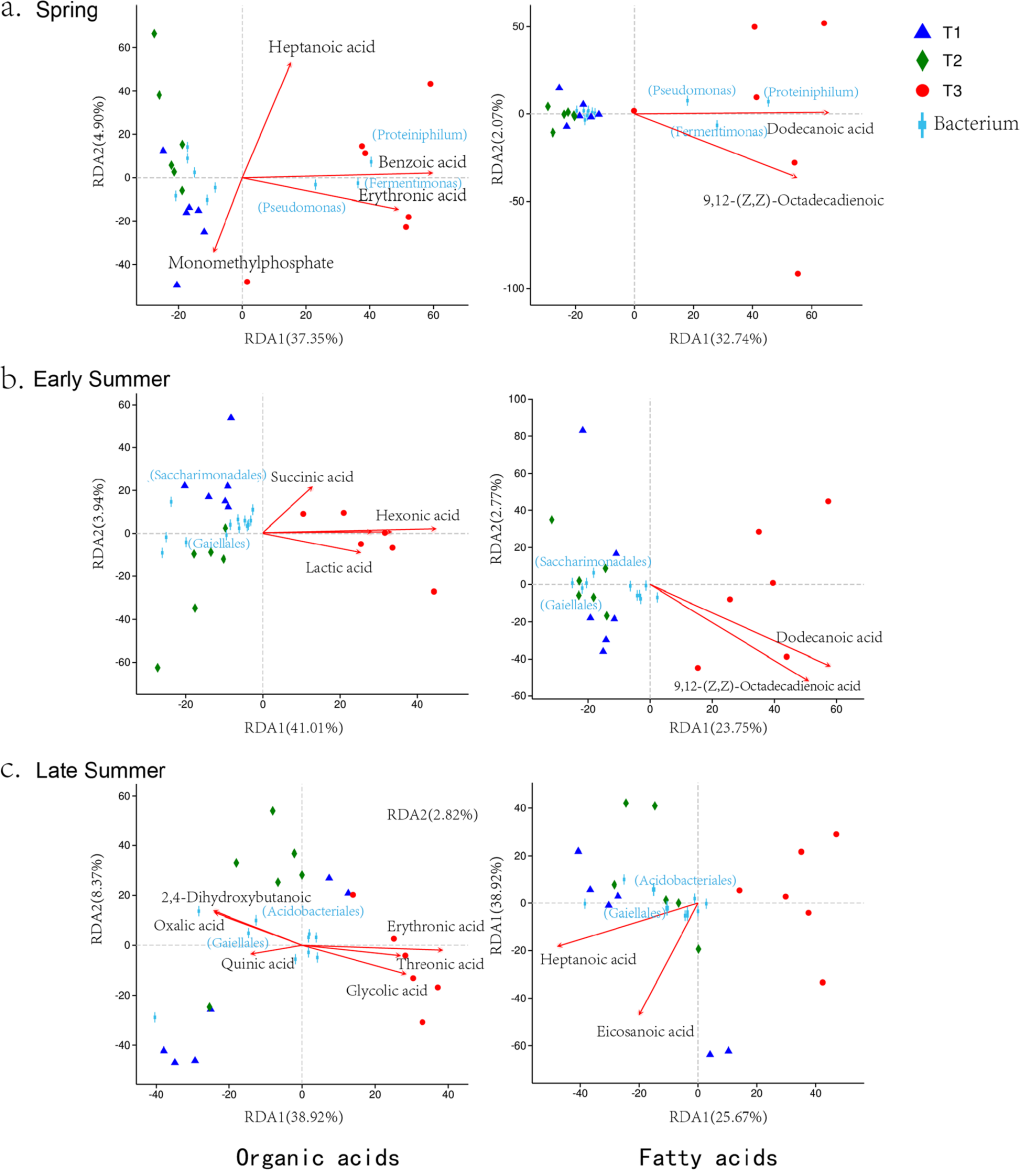
Redundancy analysis (RDA) analysis of MiSeq data and the metabolites of organic acids and fatty acids (arrows) in different growing seasons (a, spring; b, early summer; c, late summer). The values of axes 1 and 2 are the percentages explained by the corresponding axis

Based on the above analyses and the close relationship with other metabolites (Figure S3), differential metabolites classified as organic acids and fatty acids were selected for further analysis. Variance Inflation Factor (VIF) was employed to remove the VIF value of metabolites higher than 10 (Table S7). Subsequently, the relationship between the rhizosphere bacterial community composition at the genus level, and each affected metabolite was analyzed by RDA (Fig. 6) and the top-10 bacteria were selected based on abundance. The RDA revealed that most of the differential metabolites were positively affected by cow manure treatment. In spring, the abundances of *Proteiniphilum*, *Fermentimonas* and *Pseudomonas* were positively correlated to organic acids (Benzoic acid and Erythronic acid) and fatty acids (Dodecanoic acid and 9,12- (Z, Z)-Octadecadienoic). In early summer, abundances of the top-10 bacteria showed no correlation with the content of organic acids and fatty acids, and *Saccharimonadales* and *Gaiellales* showed a significantly negative correlation. In late summer, abundances of *Acidobacteriales* and *Gaiellales* depended on the variation of organic acids and fatty acids, which exhibited positive correlations with organic acids (2,4-Dihydroxybutanoic, Oxalic acid and Quinic acid) and the fatty acids (Heptanoic acid and Eicosanoic acid). They showed negative correlations with organic acids (Erythronic acid, Threonic acid and Glycolic acid).

## Discussion

Our previous study revealed that bacterial communities in the rhizosphere were influenced by both application of fertilizer/manure and the growing season. Soil pH, organic matter and available potassium were the vital environmental properties contributing to bacterial variation [11]. This study focused on how the cow manure brought more benefits based on the metabolic profiling and the relationship between microbiome and metabolites was further investigated in the rhizosphere.

### Cow manure increased the chlorophyll content of tea leaves

Plants provided carbon resources (photosynthetic products) for microbial activities in the rhizosphere through root exudates [10]. Leaf chlorophyll is a vital factor in the process of photosynthesis, which helps plants harvest light and transduce energy [12]. Chlorophyll biosynthesis was reported to be associated with the growth temperature, with the chlorophyll content being increased with the temperature rising [13]. This could explain the result that the content of chlorophyll in control and in cow manure treatment increased from spring to late summer. Interestingly, it was observed that chlorophyll content in urea treatment increased from spring to early summer but then decreased in late summer, while the content of chlorophyll in cow manure treatment increased to the maximum in late summer. The possible reason is that chlorophyll synthesis is a complex process that is influenced by many factors. Not only the temperature, the application of nitrogen fertilizer also increased leaf chlorophyll contents [14]. Cow manure released the nutrients over a longer period, which can continually supply the synthesis of chlorophyll, whereas the nitrogen in urea was probably fully exhausted in late summer resulting in decreased chlorophyll content. Moreover, the higher abundance of microorganisms affected by cow manure altered the network structure and converted the critical nutrient into a more absorbable form before assimilation by plants, which could also increase the biosynthesis of Chlorophyll in tea leaves [11].

### The effect of dominated bacteria on the rhizosphere soil

Exogenous bacterial input through the application of manures changed the soil characteristics that caused by variation in bacterial composition [15]. The bacterial phyla Proteobacteria, Bacteroidetes, Acidobacteria and Actinobacteria dominated the rhizosphere of tea plants regardless of the sampling time, which could play an important role in the plant-rhizosphere interaction. *Proteobacteria* is a major phylum of gram-negative bacteria. In the tea plantation, the soil microbiota associated with cow manure application was dominated by *Proteobacteria*, especially *Alpha-* and *Gammaprotrobacteria.* The presence of *Proteobacteria* in soils of various plant systems, such as clover, maize, soybean and grasslands, was attributed to high nutrient content [16]. Belliturk et al. (2017) reported that cow manure increased N uptake in curly lettuce [17]. In this research, the increasing abundance of *Proteobacteria* had a close relationship with the compounds classified as organic acids and fatty acids. Bolan et al. (1994) indicated that organic acids could increase the availability of soil nutrient [18]. This finding was further exemplified by a long-term fertilizer treatment study, which showed that increased nutrient availability in the soil favored growth and the abundance of *Proteobacteria* [19]. *Acidobacteria,* as one of the most abundant bacterial phyla found in terrestrial ecosystems, is involved in the degradation of plant polysaccharides [20]. Moreover, the ratio between *Proteobacteria* and *Acidobacteria* is considered an indicator of soil nutrient-content, as *Proteobacteria* were recruited in nutrient-rich soils while *Acidobacteria* were recruited in nutrient-poor soils [4, 16]. Our data provided a glimpse at these two phyla, showing the relative abundance of *Proteobacteria* was higher than *Acidobacteria* under cow manure treatment during three different sampling time (Table S3; about tenfold, threefold and threefold, respectively.). Therefore, our data showed that the plant-associated bacterial microbiota in the manured soil was enriched for species in the phylum *Proteobacteria*.

*Bacteroidetes* were much more abundant under the cow manure treatment than other treatments over the three sampling times. The role of *Bacteroidetes* in the rhizosphere has not yet been deeply elucidated, but they are known as important contributors to nutrient turnover in the rhizosphere [20, 21]. Nakayama et al. (2021) found that bacterial species belonging to *Bacteroidetes* contain genes involved in denitrification, which indicated a possible involvement in N cycling [22]. Additionally, *Actinobacteria* exhibited the same abundance order of T2 > T1 > T3 in the three different seasons. *Actinobacteria* are associated with disease-suppressive soils, which indicated that the application of cow manure may reduce soil pathogens [20].

The presence of *Cyanobacteria* and Chloroflexi in T3 samples peaked in early summer. *Cyanobacteria* inhabits moist soils and can carry out photosynthesis and N-fixation [23], and thus provide N to the colonized plant roots [24, 25]. In addition, N-fixing *Cyanobacteria* may express genes at precise stages of plant growth [20]. Our results suggest that tea plants recruited *Cyanobacteria* in early summer as demand for N supply increased, driven by the growth of new tea shoots. *Chloroflexi* was the phylum containing nitrite-oxidizing bacterium, which generally dwell in habitats enriched by high ammonium, promoting plant growth [2, 26]. These studies supported our observation to some extent that *Cyanobacteria* and *Chloroflexi* brought benefits to the development of tea plants under the cow manure treatment, especially in early summer.

### Correlation between the composition of soil microbiota and soil metabolites

Changes in developmental processes in plants resulted in changes to the composition of root metabolites exuded into the rhizosphere, in turn altering the microbial community structure [27]. As an exogenous amendment, initially, cow manure (Table S1) can affect the composition of compounds in different treatments, but it tended to be same after a long time’s degradation or transformation (Table S6). To investigate the composition and potential functions of the bacterial microbiota of the rhizosphere soil of tea plants, the metabolic pathway and the correlation between the composition of microbes and metabolites of the rhizosphere soil were analyzed over the growth period from spring to late summer. According to the metabolic pathway analysis, the enriched pathways related to sugars, amino acids and alkaloids metabolism significantly contributed to the proudly changes among the rhizosphere soils treated with different fertilizers.

Sugar metabolism is essential in the interaction between plants and rhizosphere microbes [28–30]. Fructose, maltose and trehalose in the T3 soils had a significant positive correlation with the presence of *Deinococcus-Thermus*, and there was a significant negative correlation with *Chlorobi* in late summer (Fig. 5c). This result indicated that Deinococcus*-Thermus* and *Chlorobi* can facilitate changes in sugar metabolism. That plants influence the rhizosphere microbiome is well described [20], as is the capacity of the microbiome to influence the metabolome and development of plants. Trehalose is the key molecule in the trehalose signalling network mediating the development of tea flowers [30]. The metabolome of *Arabidopsis thaliana* leaf tissues (such as sugars and sugar alcohols) was significantly changed by diverse soil microbiome treatments, which specifically increased shoot biomass without inducing changes in the root biomass [1]. It seems likely, therefore, that the growth and quality of tea were influenced by at least some degree by microbiome-generated metabolites in the rhizosphere.

Amino acids coupled with sugars synergistically regulated many pathways and cycles such as glycolysis and the citric acid cycle [31], which were involved in various activities of microbiota. The application of cow manure provided not only the C resource for bacterial activities, but also the N in the form of proteins. Some of the proteins were easily breakdown by microbiota in soil such as amino acids and short peptides that were able to be utilized by plants [32]. Moreover, the cow manure could also improve the development of tea plants by increasing the abundance of the bacteria in the surrounding soil and raising the level of chlorophyll and other beneficial factors [11], which caused the enhancement of nutrition consumption. These phenomena could explain the low concentration of alanine.

Additionally, a significant positive correlation was observed between three polyols (i.e. beta-sitosterol, campesterol and stigmasterol) and *Tenericutes* in early summer. Over 40 phytosterols, including sitosterol, campesterol and stigmasterol occurred in all higher plants, while others such as brassicasterol, were family- or species-specific [33]. Cereals and oilseed plants had significant levels of phytosterols, while levels in vegetables or nuts were considerably lower [34]. Cow manure may contain phytosterols derived from cereals or oilseed plants eaten by the animal. Previous research revealed the significant relationship between phytosterols and various bottom-up, top-down and plant primary and secondary metabolites [35]. *Tenericutes* is a phylum of bacteria that contains the class *Mollicutes*. However, the specific roles that *Tenericutes* species play in the soil ecosystem and, more specifically their roles in the rhizosphere, are little known. Our results indicated that *Tenericutes* may have a role in the synthesis of polyols.

### Correlation between rhizosphere bacteria and organic and fatty acids in the rhizosphere

Application of the fertilizer/manure treatment may mediate the composition and content of organic acids and fatty acids in the rhizosphere, thereby influencing composition of rhizosphere bacteria [36], although the specifics of the relationship between the bacterial diversity and organic and fatty acid compounds in the rhizosphere is not well understood. Organic acids are major water-soluble allelochemicals in soil and can enter root cells to influence plant growth and development. Organic acids released by roots mediated composition of bacterial and fungal communities of wheat [37]. Veach et al. (2019) demonstrated that the concentration and identity of organic acids in the rhizosphere correlated with microbial diversity [38]. In our study, rhizosphere organic acids were more abundant in late summer samples than in earlier summer, and most of them (Benzoic acid and Erythronic acid) showed a significant correlation with manure application in T3 (Fig. 6). Erythronic acid, a signalling molecule from plant roots that attracts microorganisms [39], showed positive correlations with the presence of *Fermentimonas*, *Proteiniphilum* and *Pseudomonas*, and negatively correlated with the presence of *Gaiellales* and *Saccharimonadales* in spring and late summer.

Fatty acids are key components of cellular membranes, suberin, and cutin waxes. They are of primary importance in communications between the cell and the environment [3]. The specialized triterpenes (thalianin, thalianyl fatty acid esters, and arabidin) produced by a triterpene biosynthetic network in the roots of *A. thaliana* plants modulated the profiles of its root microbiota [36]. Dodecanoic acid has activity against certain plant pathogenic fungi by reducing mycelial growth [40, 41]. Our results showed that dodecanoic acid and 9,12- (Z, Z)-octadecadienoic acid exhibited a positive correlation with the presence of *Fermentimonas*, *Proteiniphilum* and *Pseudomonas* in spring, and were negatively correlated with the presence of *Gaiellales* and *Saccharimonadales* in summer.

## Conclusions

In summary, our results reveal that manure application was the main stimulant of diversity of rhizosphere-associated bacterial communities, while the time of sampling played a smaller, yet significant role. Furthermore, the differential metabolites of soils were enriched in organic acids, fatty acids, sugars and polyols, and most of them showed positive or negative correlations with the composition of microbial communities. In particular, there was a correlation between the presence of certain soil microbes and organic acids and fatty acids in the soil. Application of manure induced apparently beneficial bacteria and metabolites. Our study showed that the application of cow manure can shape the assembly and activity of the rhizosphere bacterial community toward a higher abundance of specific rhizosphere competent bacterial taxa that may provide complementary compounds that benefit the growth of tea plants.

## Materials and methods

### Field trial

The tea plantation studied was located in Qingdao, on the northern China plain (36°19′N, 120°23′E, elevation 54.88 m). The soil at the site was classified as brown loamy soil. More than 2000 tea plants of cultivar ‘Huangshanzhong’ were growing in the plantation. Plants were eight years old and maintained at 0.45 m in height. Three different treatments were applied: T1 was unfertilized, T2 was treated with urea, N: 46.7%, and T3 was treated with composted cow manure, N: 1.5%. Each treatment had three randomly replicated blocks. Each block was 90 m^2^ under a unified management regime. In T2 and T3, the application of total N was the same (300 kg∙ha^−1^). Urea and cow manure was applied to the field plots once on the same day. Briefly, a ditch was dug to a depth of 20 cm and a width of 20 cm under the tree canopy along the root side and covered with 5 cm soil after applying the urea or cow manure. Initial soil parameters before the experiment were described in our previous paper [11]. The chemical properties of the cow manure were: organic matter (OM) 71.20%, total nitrogen (TN) 1.50%, total phosphorus (TP) 0.81% and total potassium (TK) 0.98%. The compositions of metabolites and microbes of cow manure are provided (Table S1).

### Sampling of rhizosphere soil

The rhizosphere samples in the field were collected in three different growing seasons: spring (March), early summer (June), and late summer (August) with six biological replications. Ten soil cores (0–1 cm from the root) from the middle of the fertilizer ditch were collected to a depth of 20 cm. Soil samples were homogenized. Fifty-four samples were collected randomly, and large plant residues and stones were removed by sieving. Samples were frozen quickly in liquid nitrogen and stored at -80 °C until analyses.

### Plant chlorophyll sampling

Relative contents of chlorophyll in young shoots (YS) and mature leaves (ML) were determined using a portable chlorophyll detector Micro Controller Unit (SPAD-502plus, Konica-Minolta, Japan) at each sampling time [42]. Samples of tea leaves collected from the three soil treatments (T1, T2 and T3) on the same day were named S1, S2, and S3, respectively.

### 16S rDNA amplicon sequencing analysis

The method of soil DNA extraction was as described in our previous paper [11]. Library preparation and Illumina MiSeq sequencing were done at Smart Nuclide (Suzhou city, Jiangsu province, China). DNA samples were quantified using a Qubit 2.0 Fluorometer (Invitrogen, Carlsbad, CA, USA). DNA (30–50 ng) was amplified from the V3 and V4 hypervariable regions of prokaryotic 16S rDNA. The regions were amplified using forward primers 5’- CCTACGGRRBGCASCAGKVRVGAAT-3’ and reverse primers 5’-GGACTACNVGGGTWTCTAATCC-3’ [43]. An Agilent 2100 Bioanalyzer (Agilent Technologies, Palo Alto, CA, USA) was employed to validate DNA libraries, which were multiplexed and loaded on an Illumina MiSeq instrument according to the manufacturer’s instructions (Illumina, San Diego, CA, USA). Sequencing was performed using a 2 × 300 paired-end configuration; image analysis and base calling were conducted by the MiSeq Control Software (MCS) embedded in the MiSeq instrument [43]. Analysis of 16S rRNA data was conducted using the QIIME2 data analysis package as described [30].

### GC–MS analysis

Five grams of frozen-dried rhizosphere soils were dissolved in 50 mL 80% methanol (pre-cooled at -20 °C) to extract the soil metabolites. Samples were subsequently centrifuged at 20 g at 4 °C for 10 min. Then, 100 μL of 15 mg∙mL^−1^ methoxyamine pyridine solution was added to the resulting supernatant, followed by vortexing for 30 s and incubation for 120 min at 37 °C. An aliquot of 60 μL N, O-bis-(trimethylsilyl) trifluoroacetamide (BSTFA) reagent containing 1% Trimethylchlorosilane (TMCS) was added to the mixture, then incubated for 90 min at 37 °C. Derivative samples were centrifuged at 20 g at 4 °C for 10 min. The resulting supernatant was injected into an Agilent 7890A/5975C GC–MS system (Agilent, USA) for profiling analysis. Samples were injected into the apparatus in random order. Quality control (QC) samples were used to ensure the stability of the system and monitor deviations of the analytical data. Gas chromatography was performed on a HP-5MS capillary column (5% phenyl/95% methylpolysiloxane 30 m × 250 μm i.d., 0.25 μm film thickness, Agilent J & W Scientific, Folsom, CA, USA). Six biological replicates were run twice on the GC–MS as technical repeats.

Raw GC–MS data were converted into NetCDF files by G1701 MSD ChemStation software and subsequently processed by XCMS 3.5 (www.bioconductor.org). Peak detection and deconvolution were performed with the automated mass spectral deconvolution and identification system (AMIDS) and peak lists compiled with National Institute of Standards and Technology (NIST) and Wiley libraries. The resulting data matrix was subjected to multivariate analyses and significant feature identification using MetaboAnalyst 4.0 (http://www.metaboanalyst.ca) [44].

### Statistical analysis

Leaf chlorophyll content was analyzed by the least-significant difference (*P* < 0.05) using DPS software. Other statistical analyses were performed in R software (version 3.2.1, Vienna, Austria). Hierarchical clustering analysis (HCA) and heat map were carried out using R to visualize and group metabolite profiles [45]. The Non-metric Multidimensional Scaling (NMDS) was used to visualize changes in bacterial composition [46]. Clustering of the microbiome and metabolome was performed by partial least squares-discriminant analysis (PLS-DA) and Orthogonal Projections to Latent Structures Discriminant Analysis (OPLS-DA) using Soft Independent Modeling of Class Analogy (SIMCA)-P (version 13.0, Umetrics AB, Umea, Sweden) and R package ropls. To investigate patterns of separation between microbial communities Principal Coordinate Analysis (PCoA) were calculated with Phyloseq package (v.1.10) [47]. According to pathway analysis on Metaboanalyst and KEGG metabolic database (http://www.kegg.jp/), metabolic pathway was analyzed. ANOVA was used to examine the significant differences among the different treatments with a *P*-value < 0.05 being considered as statistically significant. The redundancy analysis (RDA, " vegan" in R software) was employed to identify the relationship between bacterial community composition and metabolites in the rhizosphere [48]. The correlation of metabolite–metabolite was calculated by R language and the statistical test was adjusted by p*-*values. The corresponding p-values were also calculated using the cor.test function. Pearson correlation coefficient (*r*^*2*^≥ 0.49 and *P* ≤ 0.05) was also used to analyze the correlation between the composition of microbial communities and metabolites in the rhizosphere.

## Supplementary Information

**Addditional file 1: Figure S1.** Relative abundance (%) of the major bacterial phyla present in the soil microbial community upon different treatments. Major contributing phyla are displayed in different colors. **Figure S2.** PLS-DA score plots derived from metabolites (a) at spring, (b) early summer and (c) late summer. Each season corresponds to a soil sample and different colors indicate the field treatments. The score of variation explained by each principal component is indicated on the axes. R2X[1] and R2X[2], the degree of explanation in first component axes and the degree of explanation in second principal component axes, respectively. The t[1] and t[2], first and second principal component axes, respectively. Ellipses represent the 95% confidence regions for each sub-class of observations, assuming normal distributions. **Figure S3.** Map of significant soil metabolite-metabolite correlations (A, Spring; B, Early summer; C, Late summer). **Table S1.** The metabolic and microbial profiling of the applied cow manure. **Table S2.** OTUs of samples obtained in at the different seasons. **Table S3.** The relative abundance of the dominant phyla in soil bacterial communities. **Table S4.** The Permutational multivariate analysis of variance (PERMANOVA) among different treatments and sampling time on bacterial presence in the rhizosphere soils. **Table S5.** The abundance of Genus grouped as the phyla of *Proteobacteria *and *Actinobacteria*. **Table S6.** The soil metabolite compounds in the rhizosphere soils with three different treatments in different sampling time. **Table S7.** Variance Inflation Factor (VIF) value of organic acids and fatty acids in the rhizosphere soils in different sampling time.

## Data Availability

The datasets analyzed during the current study are available in the National Center for Biotechnology Information Short Read Archive under accession PRJNA593402 (https://www.ncbi.nlm.nih.gov/bioproject/PRJNA593402). The tea plants used in this study were provided by Tea Research Institute of Qingdao Agricultural University. The voucher specimen of tea plants is not available.

## Abbreviations

BSTFA: N, O-bis-(trimethylsilyl)trifluoroacetamide
TMCS: Trimethylchlorosilane
HCA: Hierarchical Clustering Analysis
NMDS: Non-metric Multidimensional Scaling
PCoA: Principal coordinate Analysis
PERMANOVA: Permutational Multivariate Analysis of Variance
PLS-DA: Partial Least Squares-Discriminant Analysis
RDA: Redundancy Analysis
WUF: Weighted Unifrac
UUF: Unweighted Unifrac
KEGG: Genes and Genomes database
