# Supplementary material for: Differential responses of the rhizosphere microbiome structure and soil metabolites in tea (Camellia sinensis) upon application of cow manure
Source: BMC Microbiol. 2022 Feb 14;22:55. doi: 10.1186/s12866-022-02470-9 (PMC8842532; doi:10.1186/s12866-022-02470-9)
Supplement: Supplementary file 1 — Addditional file 1: Figure S1. Relative abundance (%) of the major bacterial phyla present in the soil microbial community upon different treatments. Major contributing phyla are displayed in different colors. Figure S2. PLS-DA score plots derived from metabolites (a) at spring, (b) early summer and (c) late summer. Each season corresponds to a soil sample and different colors indicate the field treatments. The score of variation explained by each principal component is indicated on the axes. R2X[1] and R2X[2], the degree of explanation in first component axes and the degree of explanation in second principal component axes, respectively. The t[1] and t[2], first and second principal component axes, respectively. Ellipses represent the 95% confidence regions for each sub-class of observations, assuming normal distributions. Figure S3. Map of significant soil metabolite-metabolite correlations (A, Spring; B, Early summer; C, Late summer). Table S1. The metabolic and microbial profiling of the applied cow manure. Table S2. OTUs of samples obtained in at the different seasons. Table S3. The relative abundance of the dominant phyla in soil bacterial communities. Table S4. The Permutational multivariate analysis of variance (PERMANOVA) among different treatments and sampling time on bacterial presence in the rhizosphere soils. Table S5. The abundance of Genus grouped as the phyla of Proteobacteria and Actinobacteria. Table S6. The soil metabolite compounds in the rhizosphere soils with three different treatments in different sampling time. Table S7. Variance Inflation Factor (VIF) value of organic acids and fatty acids in the rhizosphere soils in different sampling time. [file 12866_2022_2470_MOESM1_ESM.zip › Supplementary figures.docx]

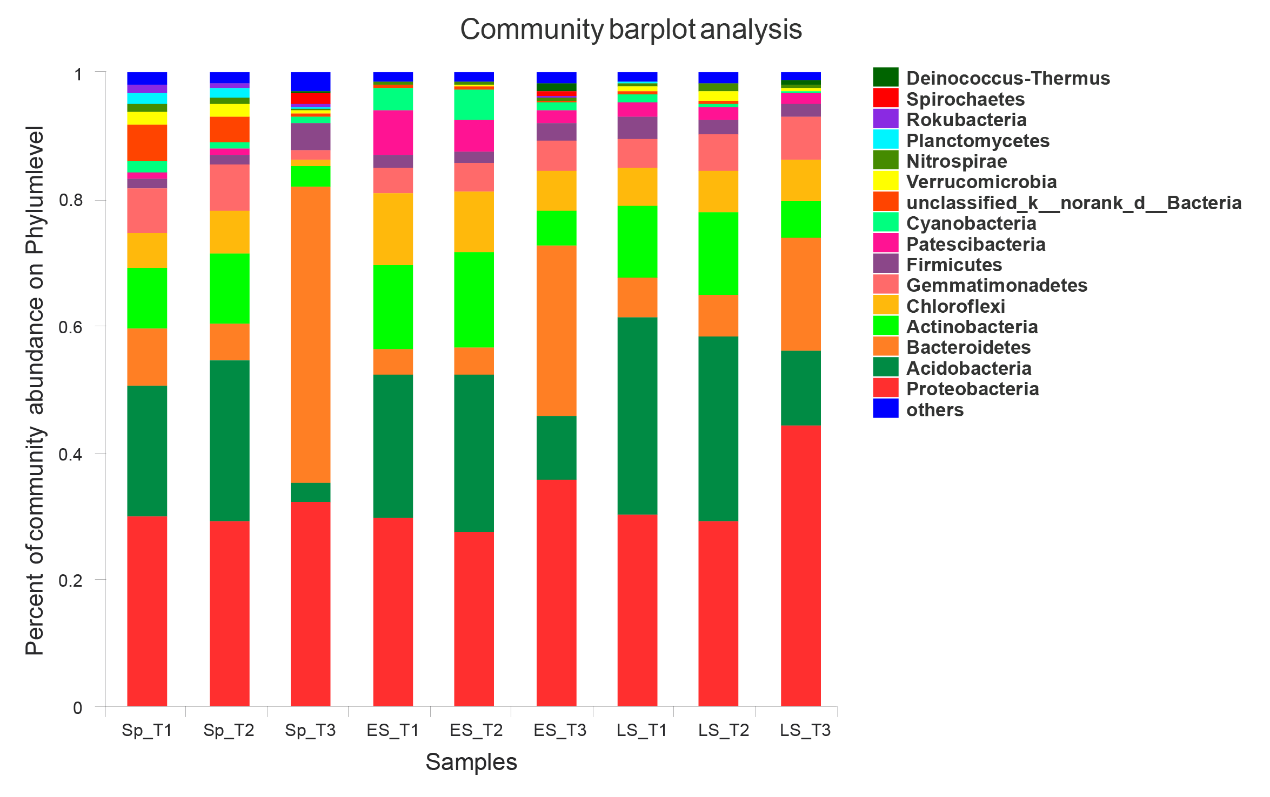


**Figure S1** Relative abundance (%) of the major bacterial phyla present in the soil microbial community upon different treatments. Major contributing phyla are displayed in different colors.


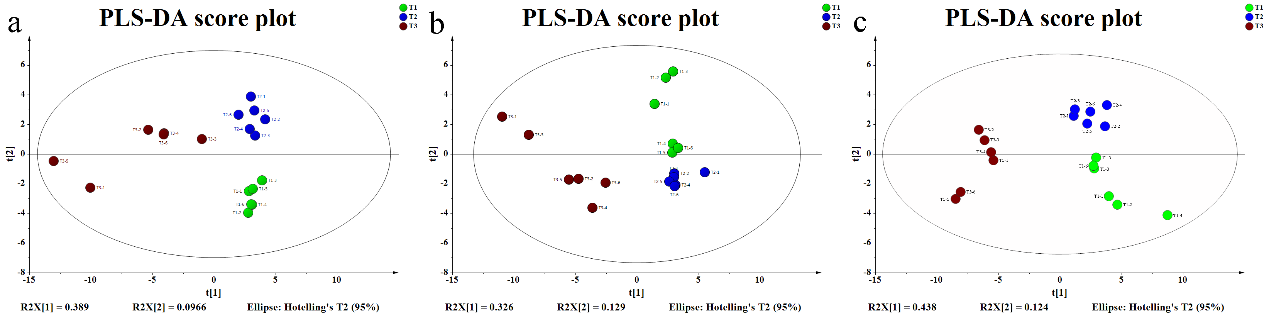


**Figure S2** PLS-DA score plots derived from metabolites (a) at spring, (b) early summer and (c) late summer. Each season corresponds to a soil sample and different colors indicate the field treatments. The score of variation explained by each principal component is indicated on the axes. R2X[1] and R2X[2], the degree of explanation in first component axes and the degree of explanation in second principal component axes, respectively. The t[1] and t[2], first and second principal component axes, respectively. Ellipses represent the 95% confidence regions for each sub-class of observations, assuming normal distributions.


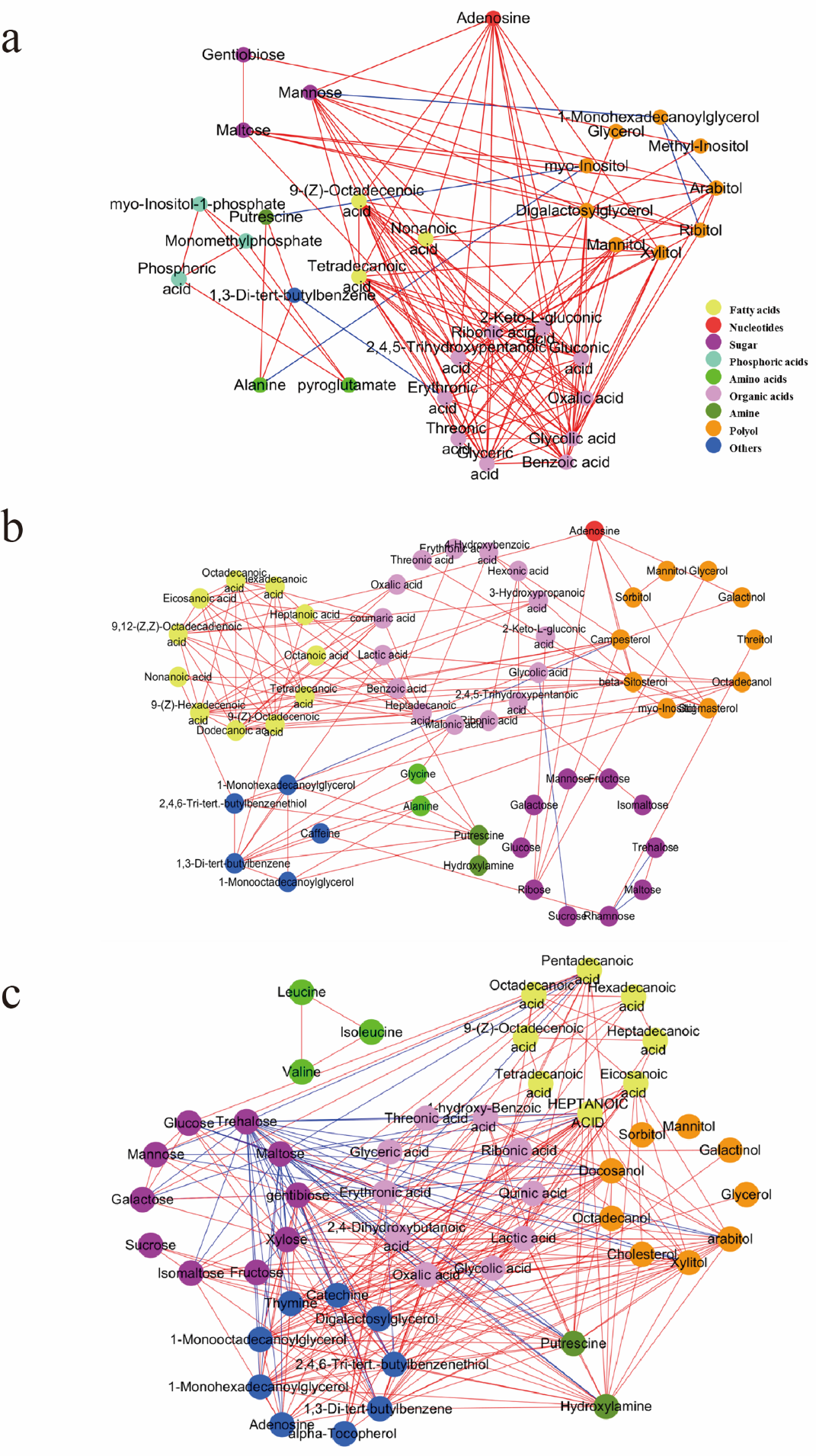


**Figure S3** Map of significant soil metabolite-metabolite correlations (a, Spring; b, Early summer; c, Late summer).
